# The Role of Nutrition in the Development, Management, and Prevention of Rheumatoid Arthritis: A Comprehensive Review

**DOI:** 10.3390/nu17243826

**Published:** 2025-12-06

**Authors:** Maria Polyzou, Andreas V. Goules, Athanasios G. Tzioufas

**Affiliations:** 1 Department of Pathophysiology, School of Medicine, Laiko General Hospital, National and Kapodistrian University of Athens, 11527 Athens, Greece; 2 Rheumazentrum Ruhrgebiet, Ruhr-University Bochum, 44649 Herne, Germany; 3 Joint Academic Rheumatology Program, Department of Pathophysiology, School of Medicine, Laiko General Hospital, National and Kapodistrian University of Athens, 11527 Athens, Greece; 4 Research Institute for Systemic Autoimmune Diseases, 11526 Athens, Greece; 5 Laboratory of Immunobiology, Center for Clinical, Experimental Surgery and Translational Research, Biomedical Research Foundation of the Academy of Athens, 11527 Athens, Greece

**Keywords:** RA, diet, nutrients, mediterranean diet, body mass index, herbs

## Abstract

**Background**: Rheumatoid arthritis (RA) is a chronic autoimmune disease, with key features being synovial hyperplasia, autoantibody production, and ultimately cartilage and bone destruction. The pathogenesis of rheumatoid arthritis (RA) is not fully understood, but it is estimated that genetic factors account for 50–60% of the risk, with the remainder attributed to environmental factors, including infectious agents, smoking, gut microbiota, and diet. Given that most current clinical trials on RA and nutrition are limited in sample size and duration, there is an unmet need for higher-quality studies in the future, a need that EULAR has already recognized. **Objective**: This article aims to investigate the impact of diet and nutritional factors on the development, progression, and potential prevention of RA. Specifically, it provides a comprehensive review of certain foods, such as alcohol, gluten, red meat, and saturated and trans fats, and their contribution to the onset and progression of rheumatoid arthritis (RA). In addition, it examines the effect of key anti-inflammatory nutrients in reducing the risk of RA, including olive oil, fatty fish, juices, and certain fruits. Finally, it discusses the potential protective effects of certain dietary patterns, such as the Mediterranean diet (MD) and diets rich in omega-3 polyunsaturated fatty acids (PUFAs). **Methods**: A comprehensive literature search was conducted in the PubMed/Medline, Science Direct, and Scopus databases (1990–2025). English-language observational studies, clinical trials, and systematic reviews addressing the relationship between diet and dietary patterns and RA were included. **Results**: High consumption of red and processed meat, saturated and trans fats, sugary drinks, and gluten (in vulnerable individuals) is associated with increased RA risk and greater disease activity, partly through pro-inflammatory pathways and gut dysbiosis. In contrast, regular intake of olive oil, fatty fish rich in omega-3 polyunsaturated fatty acids, fruit juices, cocoa, certain fruits, and vitamin D appears protective and may reduce disease activity and symptom severity. Adherence to anti-inflammatory dietary patterns, particularly the Mediterranean diet and diets rich in omega-3 fatty acids, is consistently associated with a lower incidence of RA, reduced inflammatory markers, and improved clinical outcomes. However, most available studies are limited by small sample sizes, short duration, heterogeneous methodologies, and potential confounding by other lifestyle factors (e.g., smoking, obesity). **Conclusions**: Although an appropriate diet and dietary habits cannot replace pharmacological therapy, current knowledge supports the inclusion of an anti-inflammatory diet as an adjunct strategy in the prevention and management of RA. The relatively limited studies that have been conducted suggest that high-quality, large-scale, prospective studies are needed to prevent and treat RA. These studies should incorporate genetic, microbiome, and long-term clinical endpoints, so as to establish definitive dietary recommendations and allow for personalized nutritional interventions for patients with RA.

## 1. Introduction

RA is a chronic, autoimmune, inflammatory disorder that primarily affects the joints, occasionally leading to cartilage destruction and permanent deformity and disability. It can also involve other extra-articular organs or tissues [1,2,3]. Indeed, RA displays increased morbidity and mortality rates, with a significant impact on patients’ quality of life, adversely affecting the personal and social status of patients. In recent years, the advent of targeted treatments and, therefore, the management of RA has been improved significantly. The mainstay of “targeted” strategies [4] aims to control symptomatic burden and improve patients’ quality of life and to modify disease course, preventing irreversible joint damage and future disability [5].

The etiology of RA is complex, as studies have shown that genetic, environmental, nutritional, socioeconomic, and ethnic factors contribute to the onset of the disease and response to treatment. Many of these factors act interdependently in the development and progression of the disease [2,6,7]. Gaining insights into the interaction between the aforementioned factors and potential preventive measures is anticipated to enhance the diagnosis and management of RA. The pathogenesis of RA follows a multi-step process, whereas genetic predisposition, along with environmental factors, acts synergistically to activate both the innate and adaptive immune systems, towards an autoimmune-driven tissue injury of joint synovium [6]. Recently, the role of nutrition has garnered significant attention from scientists due to its potential impact on systemic inflammation and immunomodulation. Many rheumatologists link disease onset, treatment, and overall management to dietary habits, which interact with smoking and physical exercise. An “inadequate” diet can alter the gut microbiome, leading to dysbiosis (imbalance in intestinal bacterial species), which weakens the intestinal barrier and contributes to systemic inflammation, potentially triggering or exacerbating the clinical expression of RA. In contrast, a healthy diet helps balance the gut microbiota, which produces anti-inflammatory metabolites, such as short-chain fatty acids [8]. In 2021, the European League Against Rheumatism (EULAR) recommendations highlighted how lifestyle modifications and work habits can help prevent the progression of Rheumatic and musculoskeletal diseases (RMDs), emphasizing that a balanced diet is important for patients to maintain a healthy weight and better control disease status [9].

The importance and contribution of nutrition to the onset and progression of RA are discussed in the following sections of the article, focusing on how dietary and nutritional factors may be modified accordingly to offer additional preventive measures and reinforce the beneficial effect of targeted treatments, in an attempt to introduce a more “holistic” concept for the management of RA.

## 2. Methods and Review Strategy

For the current review, relevant articles were searched in PubMed/Medline and Science Direct databases from 1990 to 2025. Articles written only in English were included in there. The search was performed in PubMed/Medline, and Science Direct databases containing the main keywords (“rheumatoid arthritis” OR “RA patients” OR “Diets in RA patients” OR “Diets in rheumatoid arthritis”) AND (“Mediterranean diet in RA patients” OR “Elimination diet in RA patients” OR “Vegan Vegetarian diet in RA” OR “Calorie restriction in RA” OR “Fasting in RA”) AND (“Natural products in rheumatoid arthritis”) OR “Beverages and RA” OR “Fasting and caloric restriction” AND “RA”. Secondary search engine, as Scopus, was also utilized.

The diversity of the human diet is particularly wide and depends on various factors, including economic status, age, and geographical region. The influence of the main human foods on the onset and progression of RA is analyzed below.

**Figure 1:**
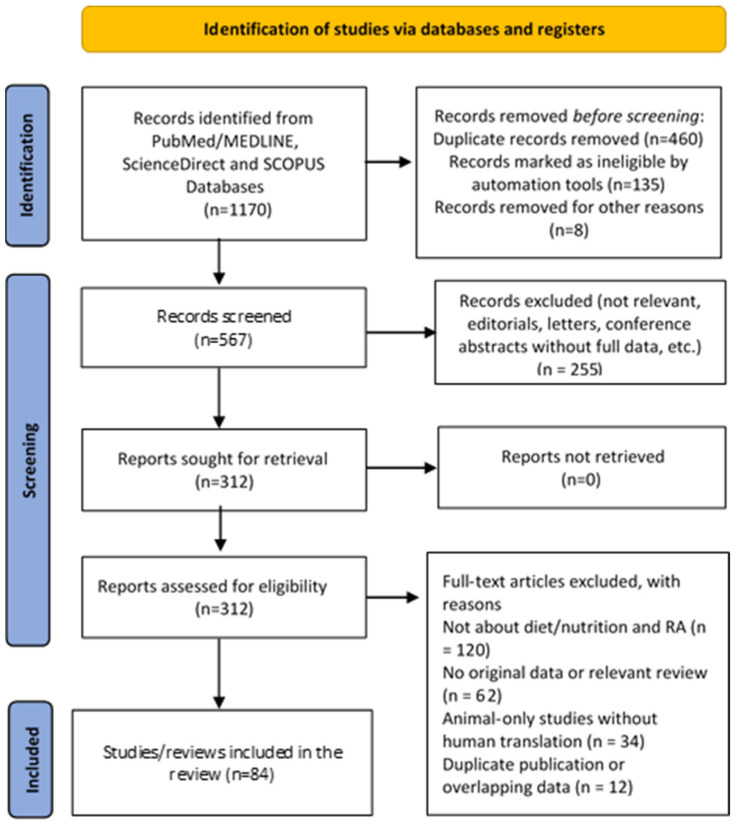
PRISMA flow chart of the study selection process.

The study selection process is illustrated in the flowchart (Figure 1), which was created according to the Preferred Reporting Items for Systematic Reviews and Meta-Analyses (PRISMA) guidelines [10].

## 3. Examining the Role of Nutrition in RA: Results

### 3.1. Beverages

A significant number of studies are examining how the nutritional content of beverages affects the onset and worsening of RA. Beverages are considered crucial in the autoimmune process of RA as they can potentially alter the gut microbiota and enhance the inflammatory pathways [11].

Some beverages are beneficial and have a positive effect on the progression of RA, while others may have a negative impact. Several underlying mechanisms have been proposed in the literature, including the anti-inflammatory effects of tea and alcohol when consumed in moderate amounts, while other beverages, such as sugary soft drinks, exhibit pro-inflammatory properties. The impact of the most basic categories of beverages on the onset and progression of RA will be discussed below.

(i). Water and juices

There is a scarcity of studies specifically linking water intake to the onset or prognosis of RA. However, the homeostatic effect through proper hydration is universally recognized as mandatory for the maintenance of good health status. Its consumption is also highly beneficial due to its rich nutrient and mineral content and is believed to potentially regulate inflammation, support joint health, and reduce the severity of arthritis flares [11].

A large body of research has investigated the effect of fruit juices on RA, especially those rich in polyphenols, which hold antioxidant and anti-inflammatory properties [12,13]. They found that custard apples improved the total antioxidant status in patients with RA. This effect was associated with the ability of fruits to suppress the production of inflammatory cells. Majeed et al. [14] experimentally studied the contribution of pineapple juice to the behavior of rats suffering from osteoarthritis or inflammatory arthritis. Pineapple juice exhibits mild anti-inflammatory effects and, therefore, was found suitable for use as a dietary adjuvant complementary to conventional anti-inflammatory medications. Nasef et al. [15] experimentally investigated the impact of Custard apple juice on reducing RA in rats.

Other studies have shown that pomegranate juice, characterized by a high concentration of polyphenols, demonstrates potent anti-atherogenic effects in both healthy human subjects and atherosclerotic mice. This benefit was most likely attributed to the significant antioxidative properties of this juice [16,17]. The effect of dietary carotenoids contained in orange juice and their antioxidant properties were studied by Pattison et al. [18], who showed that a modest increase in beta-cryptoxanthin intake contained in a glass of orange juice may reduce the risk of future development of inflammatory disorders, including RA.

Several other studies have investigated the anti-inflammatory effects of beetroot juice [19] and cranberry juice [20]. Cranberry juice appears to exert a beneficial effect on RA patients by lowering disease activity. These preliminary results require confirmation by larger, long-term trials to fully clarify the underlying mechanisms.

(ii). Coffee and tea

Coffee and tea are widely popular beverages possessing complex effects on human health. While caffeine in coffee is known to have immunomodulatory and anti-inflammatory properties, coffee consumption has been paradoxically linked to an increased risk of developing seropositive RA. In a dose–response meta-analysis conducted by Asoudeh et al. [21], five prospective cohort studies involving more than 266,000 patients were included in the analysis. The core result of the meta-analysis was a positive association between the consumption of both regular and decaffeinated coffee and an increased risk of RA. Higher overall coffee intake, more specifically one additional cup of coffee per day, was linked to 6% increase in the risk of RA. Moreover, consuming one additional cup of decaffeinated coffee per day was associated with an 11% increase in the risk of RA. This observation suggests that compounds other than caffeine, potentially related to the decaffeination process, may be responsible for this positive association. On the other hand, there was no significant association between the risk of RA and the consumption of regular coffee, tea, or total caffeine intake [21]. Asoudeh et al. [21] acknowledged that patients who drank caffeinated coffee sometimes had higher IL-6 levels compared to those consuming no coffee at all. Since IL-6 is a pro-inflammatory cytokine implicated in the pathogenesis of rheumatoid arthritis, the aforementioned finding needs to be further investigated.

Lu and Huang [22] performed a Mendelian randomization (MR) to assess the causal effect of tea intake on RA and systemic lupus erythematosus (SLE). The study concluded that MR did not support a causal link between tea intake and the development of RA and SLE.

Likewise, Dong et al. [23] found no significant association between the risk of RA and the consumption of total coffee, decaffeinated coffee, or caffeine. Moreover, the RA risk did not change drastically beyond a certain dose. Despite the neutral findings for coffee, that study suggested a positive association between tea consumption and RA risk [23]. Similarly, Westerlind et al. [24] did not find a significant link between coffee consumption and RA risk in a large population-based, case–control study involving 2184 diagnosed RA patients in Sweden [24].

In conclusion, the relationship between coffee and tea intake with RA risk remains uncertain, and further studies to confirm the potential role of coffee and tea consumption in RA are required.

(iii). Cocoa and polyphenol flavanol

Polyphenols derived from natural products have a beneficial effect in various inflammatory diseases, including RA [25]. Cocoa is a natural product that is rich in a type of polyphenol called flavanol. It appears that cocoa flavanols have significant anti-inflammatory and antioxidant activity by reducing inflammatory cytokines and reactive oxygen species (ROS), thereby increasing overall body resistance to oxidative stress. Furthermore, cocoa polyphenols have been shown to positively influence intestinal inflammation by reducing neutrophil infiltration and the production of pro-inflammatory enzymes and cytokines [26]. Although there are no in vitro or in vivo studies to support the mechanistic effects of cocoa in humans, an experimental study in female Louvain arthritic rats (LOU) fed by a cocoa-enriched diet disclosed that the cocoa-fed rats had reduced IgG2a, IgG2b, and IgG2c titers; decreased ROS production, TNFα, and NO release from peritoneal macrophages; and adjusted Th lymphocyte proportion, compared to controls; such findings suggest that a cocoa-enriched diet may serve as a good supportive therapy in disorders of autoimmune origin [27]. Long et al. [28], after evaluating existing research on the safety and efficacy of dietary polyphenols in treating RA, concluded that dietary polyphenols can reduce inflammation and oxidative stress, as evidenced clinically by improvements in DAS28. Due to the small number of studies and the heterogeneous characteristics of RA patients included in these studies, more randomized controlled trials are needed to verify the beneficial properties of dietary polyphenols.

### 3.2. Alcohol

Alcohol is one of the most consumed beverages. The negative health effects of alcohol abuse are related to diseases of the cardiovascular system, liver, and nervous system. Alcohol also exerts action on the immune system as follows: acetate, an alcohol metabolite, inhibits the function of T follicular helper (TFH) cells and antigen-presenting cells (APCs), both playing a central role in the augmentation and perpetuation of autoimmune response in RA [29]. Eventually, their suppression reduces the inflammatory and joint-destructive process. Moreover, alcohol also affects the gut microbiome and the intestinal barrier, which plays a significant role in the pathogenesis of RA [29]. There is a considerable interest in the international literature on the effect of alcohol on rheumatoid arthritis, as it appears that moderate alcohol consumption has a positive effect on disease remission [29].

Indeed, Alfredsson et al. [30] in a population-based case–control study, following 1228 drinkers and non-drinkers with newly diagnosed RA, showed that non-drinkers had both at baseline as well as at one year of follow-up, more tender and swollen joints, reported more pain and fatigue, and had a lower health-related quality of life.

Maxwell et al. [31] reported, in a study of a total of 1877 individuals (873 patients with erosive RA and 1004 healthy controls), that alcohol consumption was associated with a reduced risk for severe RA. This study also revealed an association between moderate alcohol consumption and a decreased incidence of RA. The patients were examined for RA-outcomes-measures, such as CRP, DAS28, pain visual analogue scale, modified HAQ (mHAQ), and modified Larsen score, which were all inversely correlated with a rising frequency of alcohol consumption. Alcohol use also showed a protective role for severe disease in patients with anti-CCP-antibodies and RF [31]. In a prospective study of 34.141 women who were followed up from 2003 to 2009 [32], 107 new cases of RA were recorded, and the effect of alcohol was explored. It was shown that women who drank more than four glasses of alcoholic drinks (1 glass = 15 g of ethanol) per week had a 37% reduced risk for developing RA, compared to women who drank less than 1 glass per week or who had never drunk alcohol. No significant differences regarding disease outcomes were observed among distinct alcoholic beverages, including beer, wine, and liquor, leading to the assumption that moderate consumption of alcohol, regardless of the type of drink, could exhibit a protective role in RA.

An important clinical issue is related to the use of particular medications such as methotrexate or NSAIDs (non-steroidal anti-inflammatory drugs), which, in combination with alcohol, may significantly increase the risk of liver damage (methotrexate) or gastrointestinal adverse events (NSAIDs) [4,5].

Finally, in a prospective study of 979 patients with RA (62%), psoriatic arthritis (PsA) (26.7%), and ankylosing spondylitis (11.2%) [33], it was found that heavy male drinkers (>15 units of alcohol weekly) were highly associated with increased remission rates.

### 3.3. Fruits and Herbs

According to published literature, many fruits and herbs may present beneficial effects in the course of RA. However, the mechanism of action, their quantity, and their general properties in RA are still unclear. The therapeutic potential of herbal medicine as a complementary therapeutic approach in RA is highly valued for the advantages it offers, including: (i) the natural origin, (ii) the capacity for symptom alleviation, (iii) the holistic health strategy, and (iv) the potential for personalized therapy [34,35]. Several plants and herbs are believed to offer an anti-inflammatory effect by inhibiting inflammatory mediators such as nitric oxide, IL-6, IL-1b, and various chemokines. As mentioned in the systematic review of Marquez et al. [36], plants and herbs contain potentially beneficial components such as flavonoids (luteolin), anthraquinone glycoside (Emodin), caffeic acid ester (rosmarinic acid), stilbenoid polyphenol (Resveratrol), carotenoids (cryptoxanthin, phenolic xanthonoid (mangiferin) and alkaloids (Piperlongumine). Nevertheless, robust scientific evidence supporting their efficacy is limited, and their quality and safety vary.

The role of fruits in the management of arthritis has received research attention in recent published studies. There are emerging observational data about the support role of blueberries, raspberries, and strawberries, as well as pomegranates, that may offer some protection against arthritis and negatively affect the development and progression of RA [37,38]. A meta-analysis by Dong et al. [23] showed that an increased fruit intake of 80 g/d was associated with a 5% decreased risk of RA. To conclude, several studies suggest that fruits and herbs may facilitate RA management; however, ongoing research remains essential to determine their exact mechanism of action, appropriate dosage, and general efficacy profile.

### 3.4. Omega-3

Omega-3 polyunsaturated fatty acids (PUFAs) are derived mainly from oily fish [eicosapentaenoic acid (EPA) and docosahexaenoic acid (DHA)] or plant sources [alpha-linoleic acid (ALA) [39]. EPA and DHA are believed to have potential benefits for autoimmune diseases by increasing the production of eicosanoids with anti-inflammatory activity [40]. The anti-inflammatory mechanism of fish oil has been investigated in a study, where its consumption was shown to reduce plasma levels of interleukin-1β (IL-1β) in patients with RA [41]. Along with this finding, another study showed that fish oil consumption lowered the levels of tumour necrosis factor-alpha (TNF-α), IL-1β, and interleukin-6 (IL-6) [42]. The decrease in the inflammatory cytokines by omega-3 fatty acids supports its possible beneficial effect on disease activity. Hong et al. examined the effect of omega-3 fatty acids in autoimmune diseases, including RA, in an umbrella review that included systematic reviews as well as meta-analyses. It has been shown that omega-3 fatty acids in RA can improve morning stiffness, joint pain, and swelling, as well as overall disease activity [43].

Similar results were also found for systemic lupus erythematosus (SLE). However, using the AMSTAR-2 tool, validated for clinical studies, only one study could be identified as high-quality evidence [44]. Proudman et al. [45] examined the effect of fish oil in patients with early onset RA (duration > 12 months). Patients who received simultaneously fish oil in high dose (5.5 g/day) alongside with standard triple DMARD therapy significantly reduced the likelihood of treatment failure, eliminated the need for escalation of therapy, and resulted in higher rates of ACR remission, as opposed to those patients with standard triple DMARD therapy and a low dose of fish oil (0.4 g/day); such findings may suggest that high-dose Omega-3s is an effective adjunctive intervention early in disease course. Raad et al. [39] conducted a systematic review to investigate the effect of dietary interventions, with or without omega-3 supplementation, for the treatment of RA. Diets aiming to reduce inflammation (often rich in omega-3) appear to be a promising adjunctive treatment for rheumatoid arthritis (RA), as they can help improve certain symptoms. However, researchers emphasize that we need more and larger, long-term, high-quality studies to confirm this benefit and draw definitive conclusions about the role of nutrients in disease management.

### 3.5. Vitamin D

In RA patients, Vitamin D levels are frequently low. This is attributed to the reduced outdoor activities, which lead to restricted sun exposure, the suboptimal absorption from the diet, and the inflammatory effects of the disease itself [46]. The role of Vitamin D in the immune system has been extensively studied. Apart from the role of Vitamin D in calcium and bone homeostasis, the active form of vitamin D, 1,25-dihydroxyvitamin D3 (1,25-(OH)2D3), also exhibits immunomodulatory effects. Vitamin D receptor (VDR) is widely expressed by immune cells. After activation, it suppresses pro-inflammatory T cells, such as the Th1 and Th17 lineage, and promotes T regulatory cells’ functions, enhancing the anti-inflammatory properties within the body [46,47].

In recent years, there has been an increasing interest in the role of vitamin D as a confounding risk factor for the development of autoimmune diseases, as supported by the literature. In a meta-analysis, which included fifteen studies, conducted by Lee and Bae [48], the relationship between the level of 25-hydroxyvitamin D [25(OH)D] and RA and the correlation between the level of vitamin D in serum and RA activity were evaluated. It was shown that serum vitamin D levels are markedly reduced in RA patients. Vitamin D deficiency was more common in RA patients compared to controls, and vitamin D levels were inversely correlated with RA disease activity. A significant association between VDR polymorphism and RA was also shown, suggesting that the VDR FokI F allele could potentially serve as a risk factor for RA. Similarly, another meta-analysis conducted by Lin et al. [49] showed that patients diagnosed with RA exhibited reduced Vitamin D concentrations in their serum compared to healthy controls. The data also indicated an inverse relationship between Vitamin D concentration and RA severity.

However, according to Harrison et al. [47], the key question is whether administration of Vitamin D offers greater benefits in preventing the onset of RA or can be used as a supplementary treatment for established disease.

### 3.6. Meat

The current literature has yielded conflicting results regarding the effect of meat consumption on RA. Nutrients in the form of metabolites or even metals, including arachidonic acid, nitrite, and iron, are found in several amounts in meat and are considered contributors to the inflammatory response. Indeed, arachidonic acid produces prostaglandins, which are key mediators of inflammation. Iron (Fe) might play a catalytic role in boosting the production of free radicals, and nitrite may also increase inflammation and arachidonic acid production [50].

Benino-Garcia et al. [51] explored the relationship between diet and incident RA, conducting a prospective cohort study from 1980 to 2002 in 82,063 women. Dietary intake was assessed at baseline in 1980 and on 5 subsequent occasions during follow-up. The intake of red meat, poultry, fish, and iron did not appear to be associated with an increased risk of developing rheumatoid arthritis in that cohort. Sällström et al. [52] in a small randomized controlled trial did not find any significant difference in the inflammatory response in women with or without RA, after consumption of a heavy meal with high fat and red meat. Serum IL-6 levels measured after the meal were elevated in both groups of women, matched for age and BMI. Similarly, Sundström et al. [53] found no significant association between the development of RA and the consumption of meat and meat products in a large Swedish prospective population cohort after 12 years of follow-up.

By contrast, the literature review and statistical analysis conducted by Grant et al. [50] showed that meat could be a major risk factor for the expression of RA. Similarly, Chen et al. [54], in a cross-sectional study assessing the nutritional status of Americans (NHANES) and combining data from 1999 to 2016, found a potential association between increased beef intake and the risk of RA.

Jin et al. [55] conducted a large-scale cross-sectional study from June to December 2016. Seven hundred and seven patients were enrolled and divided into two groups based on meat consumption (<100 g/day and ≥100 g/day). Meat consumption was evaluated using special questionnaires completed by the researchers. It was found that high amounts of red meat (≥100 g/day) were linked to early onset of RA, particularly among smokers and individuals with higher BMI (BMI ≥ 24 kg/m^2^). Consequently, reducing red meat intake may be recommended for patients at risk of developing RA.

### 3.7. Gluten and Casein

A gluten-free diet is considered the “treatment” for celiac disease (CD), which has also been investigated as a dietary intervention for other autoimmune diseases [56]. Furthermore, a gluten-rich diet may be potentially harmful beyond CD, leading people to exclude it from their diets. In this line, Lerner et al. [57] suggested that RA and CD share certain similarities, as they exhibit several epidemiological characteristics, environmental factors, and numerous non-HLA genetic loci in common. They are both influenced by dysbiosis and increased intestinal permeability, which probably ignites the autoimmune response that affects both the joints and the gut. In this context, Bruzzese et al. [58] demonstrated that in four RA patients with disease exacerbation who had already received conventional drug therapies, a gluten-free diet may benefit them. On the other hand, Lidon et al. [59] conducted a literature review, showing that there is insufficient scientific evidence to support a general recommendation for excluding gluten in patients with RA. Since the gluten-free diet has not been studied itself, it is too early to conclude if it has any effect on RA. The simultaneous modification of other dietary factors along with gluten exclusion makes the presence of confounding bias inevitable.

Elkan et al. (2008) [60] tried to determine if a gluten-free vegan diet could reduce cardiovascular (CVD) risk factors, which are often present in RA patients. Sixty-six patients with active RA were randomly assigned to either follow a GFVD or a well-balanced non-vegan control diet for one year. The GFVD group managed to show a statistically significant reduction in body mass index (BMI), total cholesterol, and low-density lipoprotein (LDL) cholesterol levels at 3 and 12 months after intervention. Interestingly, the levels of oxidized LDL (oxLDL), a key component in the development of atherosclerosis, were also decreased [60].

### 3.8. Curcumin

The medical use of spices like curcumin, ginger, saffron, and cinnamon dates back thousands of years in Asia. Within recent decades, modern science has also started to examine its potential benefits [61]. Sun et al. created a mouse model of medial meniscus destabilization, where they demonstrated that curcumin could decrease the expression of IL-1β, interferon-γ (IFN-γ), IL17α, IL-18, TNF-α, and vascular cell adhesion molecule 1 (VCAM1), reducing the degree of inflammation [62]. Zeng et al. [63] conducted a systematic review and meta-analysis of randomized controlled trials, with 2396 patients and 5 types of arthritis [ankylosing spondylitis (AS), rheumatoid arthritis (RA), osteoarthritis (OA), juvenile idiopathic arthritis (JIA), and gout/hyperuricemia] to estimate the role of curcumin. Curcumin and Curcuma longa extract were administered in doses ranging from 120 mg to 1500 mg for a duration of 4–36 weeks. It has been shown that curcumin and curcuma longa Extract appear to alleviate inflammation in arthritis. The results of the meta-analysis demonstrated a statistically significant difference between the rheumatoid arthritis group and the control group, showing that curcumin may reduce ESR, CRP, RF, and DAS28.

Likewise, Pourhabibi-Zarandi et al. [64] conducted a literature review, supporting the beneficial effects of curcumin on both the clinical manifestations and inflammatory markers of RA. By Contrast, Letarouilly et al. [61] in a relevant meta-analysis failed to demonstrate any beneficial effect of curcumin in RA activity. Other spices, such as garlic, ginger, cinnamon, and saffron, showed a decrease in RA activity.

### 3.9. Mediterranean Diet

It is well known that RA is expressed in a milder form in South (Mediterranean) compared to North (UK) Europe [65]. Although these differences have been attributed to immunogenetic background, dietary factors, including the Mediterranean diet, have not been investigated. Recently, the Mediterranean diet has been associated with a reduced incidence of conditions leading to chronic inflammation, in contrast to unhealthy diets, which have been linked to an increase in autoimmune diseases [66,67]. Although the influence of the Mediterranean diet on reducing the incidence of cardiovascular events, diabetes mellitus, dementia, and mortality is well known, limited data are available about its effect on RA. A cohort study conducted by Hu et al. [67] included 117,341 participants without rheumatoid arthritis who were followed up for the occurrence of RA, using the MEDI-LITE score to assess adherence to the Mediterranean diet. The results showed that higher adherence to the Mediterranean diet was associated with a lower risk for developing RA [67].

A systematic review conducted by Forsyth et al. [68] concluded that several studies have shown beneficial effects of the Mediterranean diet on reducing pain and increasing the physical activity of patients living with RA. Similarly, in another study, Hagfors et al. [69] showed that the Mediterranean Cretan Diet reduced disease severity and improved patients’ physical activity and vitality. Despite the appreciation of the beneficial effects of the Mediterranean diet in established RA, currently, there is insufficient evidence to recommend it in order to prevent RA. Studies comparing the prevalence, incidence, and clinical picture of RA in places where the Mediterranean diet predominates (e.g., South Greece—Crete, South Italy—Sicily, Israel, South Turkey, etc.) compared to northern Europe are expected to provide further insights on this issue.

Regarding the components of MD, the most basic is virgin olive oil (EVOO). Extra virgin olive oil (EVOO), maintaining its natural components, is the main source of dietary lipids in MD, which also emphasizes caloric restriction, physical activity, and a pattern rich in plant foods, fish, and moderate consumption of other animal products [66].

In a mouse model of collagen-induced arthritis (CIA), a model for RA, it was demonstrated that a diet enriched in extra virgin olive oil (EVOO) prevented the development of RA and reduced joint swelling and cartilage destruction. Mechanistically, this diet significantly reduced levels of key pro-inflammatory cytokines (TNF-α, IL-1β, and IL-17), as well as enzymes and metalloproteinases responsible for cartilage degradation (COMP and MMP-3) [70]. In conclusion, although the available research on the contribution of MD in the prevention and improvement of RA symptoms is not yet fully elucidated, most of it highlights the beneficial effects of MD in many inflammatory diseases, including RA.

### 3.10. Fasting and Caloric Restriction

RA is characterized by high morbidity due to several extra-articular manifestations and the increased cardiovascular risk, as attested by the premature atherosclerosis and the high rates of cerebrovascular events and myocardial infarction. Considering the high cardiovascular risk and the need for joint preservation, the maintenance of body weight is of paramount importance in everyday clinical practice as part of the medical advice. To achieve this target, patients limit calorie intake, either by diet modifications, increased physical activity, or fasting. The latter involves abstaining from calorie intake for specific periods (e.g., intermittent fasting), while caloric restriction (CR) refers to a reduction in calorie intake to approximately 70% of a normal diet [71]. Research data have shown that both fasting and caloric restriction should be used as complementary therapies for rheumatoid arthritis with very positive results [71,72].

Hansen et al., after careful literature review, concluded that both fasting and CR can lead to improvement of clinical symptoms such as joint pain and swelling, reduction in inflammatory markers such as C-reactive protein (CRP) and interleukin-6 (IL-6), and modification of the microbiome-related mechanisms. These results were also reflected in the improvement of the Disease Activity Score 28 (DAS28). They may also correct intestinal dysbiosis, which can cause systemic inflammation and promote autoimmune responses [71].

Even though calorie restriction (CR) can prolong both life and lifespan in many patients [73], it is noteworthy that the various psychological, social, and behavioral constraints that characterize many social groups (for example, elderly people, pregnant women) are associated with difficulties in implementing CR in real life [71].

Despite the positive findings of the research that has been carried out regarding the contribution of fasting and CR in the treatment of rheumatoid arthritis, scientists should be cautious. This is due to a lack of high-quality data, as well as the heterogeneity of the clinical studies, which may prevent the extraction of safe and reliable conclusions.

### 3.11. Maintaining Normal Body Weight

Higher body mass index (BMI) can lead to more active RA, because of the inflammatory nature of both conditions. White adipose tissue is a dynamic organ that secretes several pro-inflammatory molecules, including tumor necrosis factor (TNF)-α, interleukin (IL)-6, cytokines, and adipokines, some of which are increased in RA. Gharbia et al. [74] conducted a study in which the clinical and laboratory data, the Health Assessment Questionnaire (HAQ), and the radiographic damage score of 146 RA patients were compared at baseline, 8, 16, and 24 months. It was found that obese patients did not respond to treatment as well as overweight patients or those with normal weight, showing worse results in all measured outcomes.

Moreover, Feng et al. [75] conducted a meta-analysis, showing that a higher BMI has a positive association with RA risk, and focused on the fact that managing weight and reducing BMI could be a valuable strategy for preventing and controlling RA.

Min Son et al. [76] enrolled 335 RA patients and tried to explore the correlation between body composition (BMI, body fat mass, skeletal muscle mass) and pain, disease activity (DAS28), and disability (HAQ) in patients with RA. Obese patients displayed higher C-reactive protein levels, a higher VAS pain score, and a higher DAS28-erythrocyte sedimentation rate score. The HAQ score in females was associated with older age, a higher DAS28 score, lower skeletal muscle mass, and a higher body fat-to-skeletal muscle ratio. Likewise, Khan et al. conducted a comparative case–control observational study involving 60 patients, which showed a statistically significant correlation between body mass index and DAS-28 [77]. In a Spanish prospective, comparative, cross-sectional study of 123 patients, it was shown that obese and overweight patients had more tender and swollen joints compared to patients with normal weight [78].

Also, in other studies conducted by Lu et al. [79], Albrecht et al. [80], Stavropoulos-Kalinoglou et al. [81], and Feng et al. [75], it was supported that obese or overweight RA patients display inadequate response to treatment with poor outcomes, including a lower probability of achieving RA remission. Most studies reflect the need for further research in order to obtain reliable and safe conclusions.

Seung-Jae et al. described the contribution of obesity in the pathogenesis of RA. More specifically, they showed that the adipose tissue from both RA patients and obese animal models releases high amounts of chemokines capable of recruiting neutrophils (e.g., IL-8/MIP2, CXCL1, and CXCL5) and monocytes (e.g., IL-6, IL-1β, and CCL2) [82]. In the early phase of the disease, obesity worsens joint inflammation due to elevated IL-8/MIP2 signal, which drives an excessive number of neutrophils to migrate into the joints. In later stages, obesity participates in the maintenance and sustainability of the inflammatory process in the joints by recruiting naïve myeloid cells, which are then converted into aggressive, pro-inflammatory M1 macrophages [82].

Ah Lim et al. [83], recognizing the role of lipids in nutrition as an important source of energy, sought to investigate a possible correlation between them and the pathogenesis of rheumatoid arthritis. Lipids are essential components of cell membranes and serve as signaling messengers. They also regulate protein function through lipid-dependent post-translational modifications (e.g., myristoylation), which are crucial for protein stability and membrane localization. Lipid metabolism is dynamically regulated to support the different metabolic demands of T cells as they differentiate into either effector cells or memory cells. The key connection in RA lies in the metabolic dysregulation of these lipid-dependent pathways within the T cells. Ah Lim et al. [83] point out a functional defect in the enzyme N-myristoyltransferase, which mediates a critical lipid modification and is observed in T cells from RA patients. It obstructs the activity of AMP-activated protein kinase (AMPK) and drives the hyperactive mTOR pathway. This metabolic misdirection results in the uncontrolled differentiation of T cells into highly inflammatory and pathogenic subtypes, specifically Th1 and Th17 cells. These cells are the primary drivers of the chronic inflammation and joint destruction characteristic of RA [83].

### 3.12. Sugar and Glucose

Excessive sugar intake, especially fructose-rich sweeteners, is of great importance, as they act aggravatingly on the progression of RA through multiple pro-inflammatory pathways. Clinical observational studies consistently show that high intake of sugary drinks is associated with increased incidence of RA, disease exacerbation, and elevated CRP and IL-6 serum levels. Sugar reduction and diets low in added sugar (e.g., Mediterranean or anti-inflammatory diets) are associated with reduced pain, less morning stiffness, and lower inflammatory markers in patients with rheumatoid arthritis [84].

According to Masuko (2022) [85], the glucose metabolic pathway is significantly reprogrammed in patients with rheumatoid arthritis, directly contributing to the progression of the disease. While activated T cells typically rely on glycolysis for rapid energy and proliferation, T cells from patients with rheumatoid arthritis (RA) exhibit a shift in glucose utilization. Instead of primarily utilizing glycolysis, T cells in RA redirect glucose to the pentose phosphate pathway (PPP). This increases NADPH production, which leads to a reduction in intracellular reactive oxygen species (ROS). This shift (high NADPH/low ROS) allows T cells to hyperproliferate and adopt strongly pro-inflammatory functions, such as differentiation into pathogenic Th1 and Th17 subsets, which cause joint damage in RA. The conclusion was that impaired glucose metabolism acts as a key factor for the inflammatory phenotype observed in RA [85].

After a brief description of the impact of nutrition on RA, Table 1 is compiled. This table presents, in a general context, the main categories of nutrients mentioned above, along with their main clinical uses and health benefits, as well as the relevant mechanisms of action and diseases that are directly or indirectly related to RA.

## 4. Discussion and Future Directions

The above analysis shows that the contribution of nutrition to the pathogenesis and progression of various rheumatic diseases, including RA, is notable. Nutritional and dietary modifications cannot be characterized as a cure but can be complementary to the pharmacological treatment of RA by strengthening patients and improving their overall well-being. A diet rich in anti-inflammatory components, such as the Mediterranean diet, along with the intake of essential nutrients such as omega-3 fatty acids, antioxidants, and vitamin D, can contribute to the improvement of symptoms and the reduction in disease activity, improving patients’ quality of life. In a successful combination that includes the improvement of metabolic imbalance and modification of the intestinal microbiome, nutrition can positively impact the adaptation towards a physiological state, as well as the immune response of patients with RA.

The studies briefly analyzed in this article have shown that certain foods carry an increased risk of developing RA. Thus, the consumption of large amounts of meat, sugary drinks, combined with poor nutritional quality and obesity, is key in the development of RA. It is noteworthy that in many studies, the effect of diet on RA is combined with other factors, such as smoking or stress.

Conversely, consuming olive oil, fruit juices, cocoa, Omega-3 fatty acids, fish, and vitamin D can prevent the occurrence and improve the progression of RA. As noted by most authors, the number of studies related to the effect of various foods on RA is considered relatively limited, and therefore, the results are not particularly reliable, while recommendations for patients cannot be formulated with absolute certainty. More studies on the various dietary components are required to draw safe conclusions and increase the degree of reliability regarding their effect on the development and progression of rheumatoid arthritis.

In modern times, people’s eating habits are constantly evolving, with new dietary elements emerging alongside differentiated recommendations, highlighting the importance of continuing or increasing research on their positive or negative effects on the development and progression of rheumatoid arthritis. Upcoming data from future studies is anticipated to improve patient counseling, prevent the development of rheumatoid arthritis, and better understand the pathophysiology of the disease.

The points on which future research should focus include the following:

- Very well-structured research studies, which will consider the genetic and microbiome characteristics of patients who participate, in combination with their dietary habits;
- An efficient sample size to cover a wide clinical spectrum of RA patients and provide sufficient data with the potential for generalizability of the results.
- To understand the mechanisms through which nutrition can affect the immune response and inflammation of rheumatoid arthritis, as well as the interaction between the dietary components and the clinical picture and progression of the disease. Such insights offer the possibility for adequate and safe documentation of the effectiveness of the proposed nutritional interventions.
- To study the Interactions of RA with other associated conditions or diseases and assess the effect of nutrition on these diseases as well.
- To create conditions for personalized nutritional recommendations that will consider individuals’ genetic and immunological profiles.

## 5. Conclusions

After our analysis, the following conclusions were drawn:

- Diet is not, and therefore cannot be, characterized as a cure for rheumatoid arthritis. However, certain foods, such as the Mediterranean diet and, in general, any diet rich in omega-3, vitamin D, antioxidants, fish, and olive oil, can function as an important adjunctive therapy, since they contribute to a steady reduction in disease activity and to an improvement in quality of life.
- However, there are certain foods, such as red/processed meat, sugary drinks, and an overall poor diet—especially when combined with obesity or smoking—that significantly increase the risk and severity of RA.
- Current evidence is promising but still limited. Therefore, large, well-designed studies that incorporate genetics and the microbiome are needed before definitive, personalized dietary recommendations can be made with great certainty.

## Figures and Tables

**Table 1:** The clinical uses, the health benefits, and the relevant mechanism of action of the main nutrients.

Nutrient	Primary Clinical Uses/Health Benefits	Related Mechanism of Action/Associated Conditions	References
Water and Juices	Hydration is essential for maintaining normal bodily functions, such as temperature regulation, lubrication of joints, and kidney function, while juices provide vitamins (such as vitamin C) and minerals, which support overall health status.	Adequate water intake is vital for transporting nutrients, removing waste, and reducing the risk of kidney stones and constipation; it helps maintain cell volume and blood pressure, while juices provide immediate absorption of water-soluble vitamins (e.g., vitamin C) and electrolytes.	[11,13,14,15,20]
Coffee and tea	Both beverages are rich in antioxidants, which protect cells from damage; moderate coffee consumption reduces the risk of diseases such as Parkinson’s/Alzheimer’s disease and type 2 diabetes, while caffeine improves cognitive functions and cardiovascular health.	Improves mental alertness, concentration, and reaction time. Caffeine blocks adenosine receptors, reducing fatigue and increasing neuronal firing. Polyphenols (e.g., EGCG in tea, chlorogenic acid in coffee) act as antioxidants, reduce inflammation, and improve endothelial function.	[11,21,22,23]
Cocoa and polyphenol flavanol	Support cardiovascular health by improving blood flow and lowering blood pressure; cocoa can enhance cognitive function and memory, offer neuroprotection, and have anti-inflammatory effects.	Flavanols are compounds found in cocoa that support heart health and cognitive function by improving brain blood flow); they may reduce the risk of cardiovascular mortality and inflammation.	[26,27]
Alcohol	There is no safe level of consumption; excessive consumption can lead to addiction and liver disease; moderate consumption may be associated with a lower risk of cardiovascular disease and social anxiety.	Risk factor for many chronic diseases, including cancer and liver disease, even at mild/moderate levels. Some studies have shown that, in small amounts, it has a positive effect on heart function, reduces platelet aggregation and fibrinogen levels, and promotes NO release.	[29,32,33]
Fruits and herbs	They promote health because they are rich in nutrients and contain essential vitamins (such as A and C), minerals, and fibers; they have anti-inflammatory effects, since many fruits and herbs contain compounds that reduce inflammation and the risk of chronic diseases, such as cancer and heart disease.	They have positive effects on many conditions, such as cardiovascular disease and chronic inflammation, because they contain phytonutrients and many vitamins/minerals. Compounds such as flavonoids, carotenoids, and essential oils (in herbs) neutralize free radicals, regulate inflammatory pathways (e.g., NF-κB), and have direct antimicrobial effects.	[15,37]
Omega-3 and fatty acids	Management of hypertriglyceridemia, reduction in blood pressure and risk of heart disease, anti-inflammatory properties, support of brain health and neurodevelopment, alleviation of depression and anxiety, and improvement of cognitive functions.	They are incorporated into cell membranes and converted into anti-inflammatory (e.g., resolvins) rather than pro-inflammatory mediators; PPAR-α activation reduces hepatic triglyceride production and supports neuronal membrane fluidity; potential positive effect for treating conditions such as rheumatoid arthritis and inflammatory bowel disease (IBD).	[40,42,43]
Vitamin D	Essential for calcium absorption, osteoporosis prevention, immune function regulation; some studies suggest a link between vitamin D and mood, particularly in seasonal affective disorder (SAD).	Acting as a steroid hormone, binding to vitamin D receptor (VDR); regulation of genes involved in calcium transport (e.g., in the intestines) by modifying the function of immune cells (T-cells, macrophages).	[46,47,48,49]
Meat	Source of high-quality protein, iron, zinc, and B vitamins (especially B12); supports muscle recovery and growth, prevents anemia, especially important for active individuals.	High-quality protein containing essential amino acids, which participate in protein synthesis, enzyme function, and neurotransmitter production; a major dietary source of vitamin B12, essential for nerve function and blood formation. Excessive intake of red/processed meat is associated with an increased risk of certain types of cancer.	[50,51,54,55]
Gluten and casein	Gluten restriction is recommended for celiac disease to prevent serious gastrointestinal and systemic complications; casein found in dairy can cause digestive and inflammatory problems in people with intolerances or allergies.	In celiac disease, gluten triggers an autoimmune response that damages the intestinal villi. In allergy, casein/proteins are treated as antigens, triggering IgE-mediated inflammation.	[56,57,58]
Curcumin	Strong anti-inflammatory and antioxidant properties; it is used to manage arthritis; it improves cognitive function and reduces the risk of neurodegenerative diseases	Inhibits the main inflammatory pathways by reducing cytokine production; it increases the function of the body’s antioxidant enzymes; direct removal of free radicals	[63,64]
Mediterranean diet	Positive effect on reducing risk of cardiovascular disease, weight management, cognitive decline, and diabetes; linked to improved longevity and reduced risk of chronic diseases	Anti-inflammatory (fruits, vegetables, olive oil) and anti-oxidant (polyphenols) properties, improvement of lipid profile (healthy fats); fiber-rich diet	[8,66,68,69]
Fasting and caloric restriction	Improvement of insulin resistance and reduction in the risk of type 2 diabetes; weight management	Improves metabolic health and promotes longevity through cellular repair mechanisms. Depletes glycogen stores, forcing a shift to ketone bodies for energy.	[71]
Maintaining normal body weight	Risk reduction in diabetes, heart disease, and some forms of cancer; improvement of physical condition, mental health, and overall well-being.	Reduction in stress upon organs (heart, joints); prevention and management of osteoarthritis; normalization of insulin signaling; reduction in chronic inflammation from adipose tissue (adipokines) and improvement of hormonal balance.	[75,77,78,80,81,82,83]
Sugar and glucose	Frequent sugar consumption is associated with accelerated epigenetic aging, tooth decay, gout, and increased all-cause mortality.	At typical levels of consumption, sugar promotes de novo lipogenesis, visceral obesity, hyperuricemia, and low-grade systemic inflammation through AGE formation and activation of the NLRP3 inflammasome.	[84,85]

## Data Availability

The data underlying this article were obtained using keywords from PubMed/Medline, Science Direct and Scopus databases.

## References

[1] El-dadamony N.F., Taha M.N., Mahmoud S.F. (2018). Nutritional Status and Life Style among Rheumatoid Arthritis Clients at Zagazig University Hospitals. Zagazig Nurs. J..

[2] Mueller A.-L., Payandeh Z., Mohammadkhani N., Mubarak S.M.H., Zakeri A., Bahrami A.A., Brockmueller A., Shakibaei M. (2021). Recent Advances in Understanding the Pathogenesis of Rheumatoid Arthritis: New Treatment Strategies. Cells.

[3] Alivernini S., Firestein G., McInnes J. (2022). The pathogenesis of rheumatoid arthritis. Immunity.

[4] Smolen J.S., Aletaha D., Bijlsma J.W.J., Breedveld F.C., Boumpas D., Burmester G., Combe B., Cutolo M., de Wit M., Dougados M. (2010). Treating rheumatoid arthritis to target: Recommendations of an international task force. Ann. Rheum. Dis..

[5] Hofman Z., Roodenrijs N.M.T., Nikiphorou E., Kent A.L., Nagy G., Welsing P.M.J., van Laar J.M. (2024). Difficult-to-treat rheumatoid arthritis: What have we learned and what do we still need to learn?. Rheumatology.

[6] Butola L.-K., Anjanker A., Vagga A., Kaple M. (2018). Endogenous Factor and Pathophysiology of Rheumatoid Arthritis: An Autoimmune Disease from Decades. Int. J. Curr. Res. Rev..

[7] Jahid M., Khan K.U., Haq R.U., Ahmed R.S. (2023). Overview of Rheumatoid Arthritis and Scientific Understanding of the Disease. Mediterr. J. Rheumatol..

[8] Cutolo M., Nikiphorou E. (2022). Nutrition and Diet in Rheumatoid Arthritis. Nutrients.

[9] Gwinnutt J., Wieczorek M., Balanescu A., A Bischoff-Ferrari H., Boonen A., Cavalli G., de Souza S., de Thurah A., E Dorner T., Moe R.H. (2023). 2021 EULAR recommendations regarding lifestyle behaviours and work participation to prevent progression of rheumatic and musculoskeletal diseases. Ann. Rheum. Dis..

[10] Page M., McKenzie J., Bossuyt P., Boutron I., Hoffmann T.C., Mulrow C.D., Shamseer L., Tetzlaff J.M., Akl E.A., Brennan S.E. (2021). The PRISMA 2020 statement: An updated guideline for reporting systematic reviews. BMJ.

[11] Dey M., Cutolo M., Nikiphorou E. (2020). Beverages in Rheumatoid Arthritis: What to Prefer or to Avoid. Nutrients.

[12] Ko S.H., Choi S.W., Ye S.K., Cho B.L., Kim H.S., Chung M.H. (2005). Comparison of the antioxidant activities of nine different fruits in human plasma. J. Med. Food.

[13] Danesi F., Ferguson L.R. (2017). Could pomegranate juice help in the control of inflammatory diseases?. Nutrients.

[14] Majeed M., Borole K. (2015). Evaluation of Anti- inflammatory Effect of Pineapple Juice in Rheumatoid arthritis And Osteoarthritis Models in Rats. Int. J. Med. Health Sci..

[15] Nasef A., Yossef H., Abo El-Magd Y. (2024). Evaluation of the Anti-Rheumatic Activity of Custard Apples Fruit on Rats with Rheumatoid Arthritis Induced by Complete Freund’s Adjuvant. J. Home Econ.-Menofia Univ..

[16] Aviram M., Dornfeld L., Rosenblat M., Volkova N., Kaplan M., Coleman R. (2000). Pomegranate juice consumption reduces oxidative stress, atherogenic modifications to LDL, and platelet aggregation: Studies in humans and in atherosclerotic apolipoprotein E–deficient mice. Am. J. Clin. Nutr..

[17] Kim H., Banerjee N., Sirven M.A., Minamoto Y., Markel M.E., Suchodolski J.S., Talcott S.T., Mertens-Talcott S.U. (2017). Pomegranate polyphenolics reduce inflammation and ulceration in intestinal colitis—Involvement of the miR-145/p70S6K1/HIF1 axis in vivo and in vitro. J. Nutr. Biochem..

[18] Pattison D.J., Symmons D., Lunt M., Welch A., Bingham S., Day N., Silman A. (2005). Dietary-cryptoxanthin and inflammatory polyarthritis: Results from a population-based prospective study. Am. J. Clin. Nutr..

[19] Assimiti D. (2019). The Use of Beetroot as Natural Solutions for Reducing Inflammation—Case Studies from Thailand (P12-046-19). Curr. Dev. Nutr..

[20] Thimóteo N.S.B., Iryioda T.M.V., Alfieri D.F., Rego B.E.F., Scavuzzi B.M., Fatel E., Lozovoy M.A.B., Simão A.N.C., Dichi I. (2019). Cranberry juice decreases disease activity in women with rheumatoid arthritis. Nutrition.

[21] Asoudeh F., Dashti F., Jayedi A., Hemmati A., Fadel A., Mohammadi H. (2022). Caffeine, Coffee, Tea and Risk of Rheumatoid Arthritis: Systematic Review and Dose-Response Meta-analysis of Prospective Cohort Studies. Front. Nutr..

[22] Lu R.-B., Huang J. (2023). Testing relationship between tea intake and the risk of rheumatoid arthritis and systemic lupus erythematosus: A Mendelian randomization study. Adv. Rheumatol..

[23] Dong Y., Webster J., Uzokwe C., Greenwood D., Hardie L., Cade J. (2023). Associations of tea, coffee, and caffeine intake on rheumatoid arthritis risk: A dose-response meta-analysis of cohort studies. Proc. Nutr. Soc..

[24] Westerlind H., Dukuzimana J., Lu X., Alfredsson L., Klareskog L., Di Giuseppe D. (2022). Investigation of the association between coffee and risk of RA—Results from the Swedish EIRA study. Arthritis Res. Ther..

[25] Sung S., Kwon D., Um E., Kim B. (2019). Could Polyphenols Help Control Rheumatoid Arthritis?. Molecules.

[26] Andujar I., Recio M.C., Giner R.M., Rıos J.L. (2012). Cocoa Polyphenols and Their Potential Benefits for Human Health. Oxidative Med. Cell. Longev..

[27] Ramos-Romero S., Perez-Cano F., Perez-Berezo T., Castellote C., Franch A., Castell M. (2012). Effect of a cocoa flavonoid-enriched diet on experimental autoimmune arthritis. Br. J. Nutr..

[28] Long Z., Xiang W., He Q., Xiao W., Wei H., Li H., Guo H., Chen Y., Yuan M., Yuan X. (2023). Efficacy and safety of dietary polyphenols in rheumatoid arthritis: A systematic review and meta-analysis of 47 randomized controlled trials. Front. Immunol..

[29] Azizov V., Zaiss M.M. (2021). Alcohol Consumption in Rheumatoid Arthritis: A Path through the Immune System. Nutrients.

[30] Alfredsson L., Klareskog L., Hedström K. (2023). Disease Activity and Health-Related Quality of Life Among Patients With Rheumatoid Arthritis With Different Alcohol Consumption Habits. Arthritis Rheumatol..

[31] Maxwell A., Zapien M., Pearce G., MacCallum G., Stone P. (2002). Randomized Trial of a Medical Food for the Dietary Management of Chronic, Stable Angina. J. Am. Coll. Cardiol..

[32] Di Giuseppe D., Alfredsson L., Bottai M., Askling J., Wolk A. (2012). Long term alcohol intake and risk of rheumatoid arthritis in women: A population based cohort study. BMJ.

[33] Turk M., Murray K., Alammari Y., Gorman A., Young F., Gallagher P., Saber T., Freeman L., Maguire S., O’shea F. (2021). The effects of alcohol consumption and its associations with disease activity among 979 patients with inflammatory arthritis. RMD Open.

[34] Jyoti B., Wadekar J., Sawant R., Patel U. (2015). Rheumatoid arthritis and herbal drugs: A review. J. Phytopharm..

[35] Singh A., Kaushik M., Sinha S., Oraon R.K.K., Sharma N., Rautela I. (2023). Herbal Allies for Rheumatoid Arthritis: A Comprehensive Review of Natural Products. Plant Sci. Today.

[36] Marquez A.-M., Evans C., Boltson K., Kesselman M. (2020). Nutritional interventions and supplementation for rheumatoid arthritis patients: A systematic review for clinical application, Part 3: Fruits and Herbs. Curr. Rheumatol. Res..

[37] Basu A., Schell J., Scofield H. (2018). Dietary fruits and arthritis. Food Funct..

[38] Backlund R.-T., Drake I., Bergstrom U., Compagno M., Sonestedt E., Turesson C. (2024). Adherence to dietary guidelines, and the risk of developing rheumatoid arthritis: Results from a nested case-control study. Rheumatology.

[39] Raad T., Griffin A., George E.S., Larkin L., Fraser A., Kennedy N., Tierney A.C. (2021). Dietary Interventions with or without Omega-3 Supplementation for the Management of Rheumatoid Arthritis: A Systematic Review. Nutrients.

[40] Kostoglou-Athanassiou I., Athanassiou L., Athanassiou P. (2020). The Effect of Omega-3 Fatty Acids on Rheumatoid Arthritis. Mediterr. J. Rheumatol..

[41] Espersen G.T., Ngrunnet N., Lervang H.H., Nielsen G.L., Thomsen B.S., Faarvang K.L., Dyerberg J., Ernst E. (1992). Decreased interleukin-1 beta levels in plasma from rheumatoid arthritis patients after dietary supplementation with n-3 polyunsaturated fatty acids. Clin. Rheumatol..

[42] Caughey G.E., Mantzioris E., Gibson R.A., Cleland L.G., James M.J. (1996). The effect on human tumor necrosis factor alpha and interleukin 1 beta production of diets enriched in n-3 fatty acids from vegetable oil or fish oil. Am. J. Clin. Nutr..

[43] Hong K. (2024). Association between Omega-3 fatty acids and autoimmune disease: Evidence from the umbrella review and Mendelian randomization analysis. Autoimmun. Rev..

[44] Senftleber N.K., Nielsen S.M., Andersen J.R., Bliddal H., Tarp S., Lauritzen L., Furst D.E., Suarez-Almazor M.E., Lyddiatt A., Christensen R. (2017). Marine oil supplements for arthritis pain: A systematic review and Meta-analysis of randomized trials. Nutrients.

[45] Proudman S., James M.J., Spargo L.D., Metcalf R.G., Sullivan T.R., Rischmueller M., Flabouris K., Wechalekar M.D., Lee A.T., Cleland L.G. (2015). Fish oil in recent onset rheumatoid arthritis: A randomised, double-blind controlled trial within algorithm-based drug use. Ann. Rheum. Dis..

[46] Holick M. (2011). Vitamin D: A D-Rightful Solution for Health. J. Investig. Med..

[47] Harrison S., Li D., Jeffery L., Raza K., Hewison M. (2020). Vitamin D, Autoimmune Disease and Rheumatoid Arthritis. Calcif. Tissue Int..

[48] Lee Y.H., Bae S.-C. (2016). Vitamin D level in rheumatoid arthritis and its correlation with the disease activity: A meta-analysis. Clin. Exp. Rheumatol..

[49] Lin J., Liu J., Davies M.L., Chen W. (2016). Serum Vitamin D Level and Rheumatoid Arthritis Disease Activity: Review and Meta-Analysis. PLoS ONE.

[50] Grant W. (2000). The role of meat in the expression of rheumatoid arthritis. Br. J. Nutr..

[51] Benito-Garcia E., Feskanich D., Hu F., Mandl L., Karlson E. (2007). Protein, iron, and meat consumption and risk for rheumatoid arthritis: A prospective cohort study. Arthritis Res. Ther..

[52] Sallstrom T., Barebring L., Hulander E., Gjertsson I., Winkvist A., Lindqvist H. (2025). Inflammatory and lipemic response to red meat intake in women with and without Rheumatoid Arthritis: A single meal study within a randomized controlled trial. BMC Nutr..

[53] Sundström B., Ljung L., Di Giuseppe D. (2019). Consumption of Meat and Dairy Products Is Not Associated with the Risk for Rheumatoid Arthritis among Women: A Population-Based Cohort Study. Nutrients.

[54] Chen W., Liu K., Huang L., Mao Y., Wen C., Ye D., He Z. (2022). Beef intake and risk of rheumatoid arthritis: Insights from a cross-sectional study and two-sample Mendelian randomization. Front. Nutr..

[55] Jin J., Li J., Gan Y., Liu J., Zhao X., Chen J., Zhang R., Zhong Y., Chen X., Wu L. (2021). Red meat intake is associated with early onset of rheumatoid arthritis: A cross-sectional study. Sci. Rep..

[56] El-Chammas K., Danner E. (2011). Gluten-Free Diet in Nonceliac Disease. Nutr. Clin. Pract..

[57] Lerner B., Green P., Lebwohl B. (2019). Going Against the Grains: Gluten-Free Diets in Patients Without Celiac Disease—Worthwhile or Not?. Dig. Dis. Sci..

[58] Bruzzese V., Scolieri P., Pepe J. (2020). Efficacy of gluten-free diet in patients with rheumatoid arthritis. Reumatismo.

[59] Lidón A.-C., Patricia M.-L., Vinesh D., Marta M.-S. (2022). Evaluation of Gluten Exclusion for the Improvement of Rheumatoid Arthritis in Adults. Nutrients.

[60] Elkan A.C., Sjöberg B., Kolsrud B., Ringertz B., Hafström I., Frostegård J. (2008). Gluten-free vegan diet induces decreased LDL and oxidized LDL levels and raised atheroprotective natural antibodies against phosphorylcholine in patients with rheumatoid arthritis: A randomized study. Arthritis. Res. Ther..

[61] Letarouilly J.G., Sanchez P., Nguyen Y., Sigaux J., Czernichow S., Flipo R.-M., Sellam J., Daïen C. (2020). Efficacy of Spice Supplementation in Rheumatoid Arthritis: A Systematic Literature Review. Nutrients.

[62] Sun Y., Liu W., Zhang H., Li H., Liu J., Zhang F., Jiang T., Jiang S. (2017). Curcumin Prevents Osteoarthritis by Inhibiting the Activation of Inflammasome NLRP3. J. Interf. Cytok. Res..

[63] Zeng L., Yang T., Yang K., Yu G., Li J., Xiang W., Chen H. (2022). Efficacy and Safety of Curcumin and Curcuma longa Extract in the Treatment of Arthritis: A Systematic Review and Meta-Analysis of Randomized Controlled Trial. Front Immunol..

[64] Pourhabibi-Zarandi F., Shojaei-Zarghani S., Rafraf M. (2021). Curcumin and rheumatoid arthritis: A systematic review of literature. Int. J. Clin. Pract..

[65] Drosos A., Lanchbury J.S., Panayi G.S., Moutsopoulos H.M. (1992). Rheumatoid Arthritis in Greek and British Patients. A comparative clinical, radiologic, and serologic study. Arthritis Rheum..

[66] Aparicio-Soto M., Sánchez-Hidalgo M., Ángeles Rosillo M., Luisa Castejón M., Alarcón-De-La-Lastra C. (2016). Extra virgin olive oil: A key functional food for prevention of immune-inflammatory diseases. Food Funct..

[67] Hu P., Lee E.K.-P., Li Q., Tam L.-S., Wong S.Y.-S., Poon P.K.-M., Yip B.H.-K. (2025). Mediterranean diet and rheumatoid arthritis: A nine-year cohort study and systematic review with meta-analysis. Eur. J. Clin. Nutr..

[68] Forsyth C., Kouvari M., D’cUnha N.M., Georgousopoulou E.N., Panagiotakos D.B., Mellor D.D., Kellett J., Naumovski N. (2018). The effects of the Mediterranean diet on rheumatoid arthritis prevention and treatment: A systematic review of human prospective studies. Rheumatol. Int..

[69] Hagfors L. (2005). Mediterranean dietary intervention study of patients with rheumatoid arthritis. Scand. J. Nutr..

[70] Rosillo M.A., Sánchez-Hidalgo M., Sánchez-Fidalgo S., Aparicio-Soto M., Villegas I., Alarcón-de-la-Lastra C. (2016). Dietary extra-virgin olive oil prevents inflammatory response and cartilage matrix degradation in murine collagen-induced arthritis. Eur. J. Nutr..

[71] Hansen B., Sánchez-Castro M., Schintgen L., Khakdan A., Schneider J.G., Wilmes P. (2025). The impact of fasting and caloric restriction on rheumatoid arthritis in humans: A narrative review. Clin. Nutr..

[72] Coradduzza D., Bo M., Congiargiu A., Azara E., De Miglio M.R., Erre G.L., Carru C. (2023). Decoding the Microbiome’s Influence on Rheumatoid Arthritis. Microorganisms.

[73] Hofer S., Carmona-Gutierrez D., Mueller M., Madeo F. (2022). The ups and downs of caloric restriction and fasting: From molecular effects to clinical application. EMBO Mol. Med..

[74] Gharbia O.M., El-Bahnasawy A.S., Okasha A.E., Abd El-Karim S.A. (2018). Impact of obesity on rheumatoid arthritis: Relation with disease activity, joint damage, functional impairment and response to therapy. Int. J. Clin. Rheumatol..

[75] Feng X., Xu X., Shi Y., Liu X., Liu H., Hou H., Ji L., Li Y., Wang W., Wang Y. (2019). Body Mass Index and the Risk of Rheumatoid Arthritis: An Updated Dose-Response Meta-Analysis. BioMed Res. Int..

[76] Min Son K., Hun Kang S., Il Seo Y., Ah Kim H. (2021). Association of body composition with disease activity and disability in rheumatoid arthritis. Korean J. Intern. Med..

[77] Khan F., Aziz W., Farrukh S., Rasheed U., Zammarrud S. (2021). Correlation of Body Mass Index with Disease Activity in Rheumatoid Arthritis. Ann. Pak. Inst. Med. Sci..

[78] Alvarez-Nemegyei J., Pacheco-Pantoja E., González-Salazar M., López-Villanueva R.F., May-Kim S., Martínez-Vargas L., Quintal-Gutiérrez D. (2020). Association between overweight/obesity and clinical activity in rheumatoid arthritis. Rheumatol. Clin..

[79] Lu B., Rho Y.H., Cui J., Iannaccone C.K., Frits M.L., Karlson E.W., Shadick N.A. (2014). Associations of Smoking and Alcohol Consumption with Disease Activity and Functional Status in Rheumatoid Arthritis. J. Rheumatol..

[80] Albrecht K., Richter A., Callhoff J., Huscher D., Schett G., Strangfeld A., Zink A. (2016). Body mass index distribution in rheumatoid arthritis: A collaborative analysis from three large German rheumatoid arthritis databases. Arthritis Res. Ther..

[81] Stavropoulos-Kalinoglou A., Metsios G., Koutedakis Y., Kitas G. (2016). Body-size phenotypes and cardiometabolic risk in Rheumatoid Arthritis. Mediterr. J. Rheumatol..

[82] Seung-Jae K., Chen Z., Essani A.B., A Elshabrawy H., Volin M.V., Fantuzzi G., McInnes I.B., Baker J.F., Finn P., Kondos G. (2017). Differential impact of obesity on the pathogenesis of RA or preclinical models is contingent on the disease status. Ann. Rheum. Dis..

[83] Ah Lim S., Su W., Chapman N.M., Chi H. (2022). Lipid metabolism in T cell signaling and function. Nat. Chem. Biol..

[84] Hu Y., Costenbader K.H., Gao X., Al-Daabil M., A Sparks J., Solomon D.H., Hu F.B., Karlson E.W., Lu B. (2014). Sugar-sweetened soda consumption and risk of developing rheumatoid arthritis in women. Am. J. Clin. Nutr..

[85] Masuko K. (2022). Glucose as a Potential Key to Fuel Inflammation in Rheumatoid Arthritis. Nutrients.

